# Identification of lncRNA–miRNA–mRNA Networks Linked to Non-small Lung Cancer Resistance to Inhibitors of Epidermal Growth Factor Receptor

**DOI:** 10.3389/fgene.2021.758591

**Published:** 2021-11-12

**Authors:** Ting Wang, Chengliang Yang, Bing Li, Ying Xing, Jian Huang, Yangping Zhang, Shanshan Bu, Hong Ge

**Affiliations:** ^1^ Department of Radiation Oncology, The Affiliated Cancer Hospital of Zhengzhou University, Zhengzhou, China; ^2^ The Fourth Department of Medical Oncology, Harbin Medical University Cancer Hospital, Harbin, China

**Keywords:** EGFR-TKI, resistance, NSCLC, competitive endogenous RNA, network

## Abstract

**Background:** Tyrosine kinase inhibitors that act against epidermal growth factor receptor (EGFR) show strong efficacy against non-small cell lung cancer (NSCLC) involving mutated EGFRs. However, most such patients eventually develop resistance to EGFR-TKIs. Numerous researches have reported that messenger RNAs (mRNAs) and non-coding RNAs (ncRNAs) may be involved in EGFR-TKI resistance, but the comprehensive expression profile and competitive endogenous RNA (ceRNA) regulatory network between mRNAs and ncRNAs in EGFR-TKI resistance of NSCLC are incompletely known. We aimed to define a ceRNA regulatory network linking mRNAs and non-coding RNAs that may mediate this resistance.

**Methods:** Using datasets GSE83666, GSE75309 and GSE103352 from the Gene Expression Omnibus, we identified long non-coding RNAs (lncRNAs), microRNAs (miRNAs) and mRNAs differentially expressed between NSCLC cells that were sensitive or resistant to EGFR-TKIs. The potential biological functions of the corresponding differentially expressed genes were analyzed based KEGG pathways. We combined interactions among lncRNAs, miRNAs and mRNAs in the RNAInter database with KEGG pathways to generate transcriptional regulatory ceRNA networks associated with NSCLC resistance to EGFR-TKIs. Kaplan-Meier analysis was used to assess the ability of core ceRNA regulatory sub-networks to predict the progression-free interval and overall survival of NSCLC. The expression of two core ceRNA regulatory sub-networks in NSCLC was validated by quantitative real-time PCR.

**Results:** We identified 8,989 lncRNAs, 1,083 miRNAs and 3,191 mRNAs that were differentially expressed between patients who were sensitive or resistant to the inhibitors. These DEGs were linked to 968 biological processes and 31 KEGG pathways. Pearson analysis of correlations among the DEGs of lncRNAs, miRNAs and mRNAs identified 12 core ceRNA regulatory sub-networks associated with resistance to EGFR-TKIs. The two lncRNAs ABTB1 and NPTN with the hsa-miR-150–5p and mRNA SERPINE1 were significantly associated with resistance to EGFR-TKIs and survival in NSCLC. These lncRNAs and the miRNA were found to be down-regulated, and the mRNA up-regulated, in a resistant NSCLC cell line relative to the corresponding sensitive cells.

**Conclusion:** In this study, we provide new insights into the pathogenesis of NSCLC and the emergence of resistance to EGFR-TKIs, based on a lncRNA-miRNA-mRNA network.

## Background

Lung cancer is a major cause of malignant tumor-related deaths worldwide, accounting for one quarter of all cancer deaths (Siegel et al., 2021). Non-small cell lung cancer (NSCLC), which is the main cause of patient morbidity and mortality from lung cancer, accounts for 85% of all lung tumors (Duma et al., 2019). Tyrosine kinase inhibitors that act against the epidermal growth factor receptor (EGFR-TKIs) have shown clinical benefit against NSCLC involving EGFR mutations (Mok et al., 2009; Wu et al., 2014). However, most patients eventually develop resistance and suffer disease progression (Maemondo et al., 2010). Drug resistance is an important barrier to cancer effectively treated, but the molecular mechanisms behind NSCLC resistance to EGFR-TKIs are largely unknown.

Long noncoding RNAs (lncRNAs), ranging in length from 200 nucleotides to 100 kb, regulate target gene expression at the transcriptional and post-transcriptional levels (Novikova et al., 2013). In the past years, it has been revealed that the dysregulated lncRNA profile is closely linked to tumor growth and metastasis, and numerous lncRNAs have been identified as potential clinical biomarkers and therapeutic targets in cancer (Pichler et al., 2020; Xiao et al., 2020; Yuan et al., 2020). In fact, dysregulation of lncRNAs has been linked to NSCLC resistance to EGFR-TKIs (Chen et al., 2020a; Huang et al., 2020a). For instance, such resistance has been associated with up-regulation of the lncRNA BLACAT1, and down-regulating this lncRNA restores sensitivity to EGFR-TKIs (Shu et al., 2020).

One way in which lncRNAs can regulate target gene expression is by “sponging” microRNAs (miRNAs), which are 18–25 nt long. By binding to miRNAs, lncRNAs prevent them from binding to the 3′‐untranslated region of their target mRNAs, thereby preventing the miRNAs from down-regulating target gene expression (Wang et al., 2010; Salmena et al., 2011). Indeed, networks of lncRNAs, miRNAs and mRNAs appear to be important in the pathogenesis of NSCLC and emergence of resistance to EGFR-TKIs (Li et al., 2018; Liu et al., 2019; Ma et al., 2019). For instance, down-regulation of the lncRNA RHPN1-AS1 ultimately render NSCLC cells resistant to gefitinib by targeting miR-299–3p/TNFSF12 pathway (Li et al., 2018). These single-pathway studies indicate the need for a comprehensive analysis of the network of interacting lncRNAs, miRNAs and mRNAs in NSCLC that are related to EGFR-TKI resistance. The competing endogenous RNA (ceRNA) hypothesis was considered as a new regulatory mechanism between noncoding RNAs and coding RNAs (Salmena et al., 2011). CeRNAs are transcripts that can regulate each other at post-transcription level by acting as endogenous molecular sponges to bind to competing for miRNAs. Accumulating evidence have indicated this complex crosstalk of the ceRNA network in NSCLC( (Liu et al., 2021), (Wang et al., 2020)).This means searching for the network of “competing endogenous” RNA (ceRNA) that act via shared miRNA targets to regulate expression of proteins related to EGFR-TKI resistance.

Here we developed a ceRNA network linking lncRNAs, miRNAs and mRNAs in NSCLC in an effort to understand pathways mediating resistance to EGFR-TKIs. In this study, we adopted bioinformatic methods to identify differentially expressed lncRNAs, miRNAs and mRNAs, and constructed lncRNA-miRNA-mRNA ceRNA networks involved in NSCLC resistance to EGFR-TKIs. 12 core ceRNA regulatory sub-networks, we identified two ceRNA regulatory sub-networks that interactors may be linked to resistance to EGFR-TKIs in NSCLC cells and to survival in NSCLC. Two lncRNAs, ABTB1 and NPTN, that interact with the miRNA hsa-miR-150–5p and the mRNA SERPINE1.

## Materials and Methods

### Data Sources

All genomic and clinical data were obtained from the Gene Expression Omnibus (GEO) database under accession numbers GSE83666, GSE75309 and GSE103352. To evaluate the impact of ceRNA networks expression on the survival of NSCLC patients, another lncRNA, mRNA and miRNA expression data set of NSCLC samples was downloaded from The Cancer Genome Atlas (TCGA) database.

### Profiles of lncRNA, miRNA, and mRNA Expression

Data on microarray analyses of lncRNA, miRNA and mRNA expression in NSCLC cells from patients, which were resistant or sensitive to EGFR-TKIs were obtained from the GEO database. All expression data were standardized and analyzed using limma (Ritchie et al., 2015). We identified lncRNAs, miRNAs and mRNAs differentially expressed between sensitive or resistant cancer, based on the criteria of -log10 (fold change) < 0.01 and *p* < 0.05.

### Analysis of Enrichment Functions and Pathways

Differentially expressed lncRNAs, miRNAs and mRNAs were analyzed based on Gene Ontology (GO) and Kyoto Encyclopedia of Genes and Genomes (KEGG) pathways using clusterProfiler. Enrichment was defined as *p* < 0.05 (Yu et al., 2012).

### Prediction of Targets of miRNAs and lncRNAs

Interactions between differentially expressed miRNAs and mRNAs in NSCLC resistant to EGFR-TKIs were identified by screening the RNAInter database (Lin et al., 2020). And, 104,857,6 interactions pairs are extracted from the database. A similar procedure was followed to identify interactions between differentially expressed lncRNAs and miRNAs.

### Construction of lncRNA–miRNA–mRNA Regulatory Networks

The lncRNA-miRNA pairs and miRNA-mRNA pairs, identified as described above, were merged together into a ceRNA network based on lncRNAs, miRNAs and mRNAs related to NSCLC resistance to EGFR-TKIs. Pearson correlation analysis was carried out among the species in the network, and interactions associated with a correlation coefficient R > 0.75 and *p* < 0.01 were considered reliable. The potential network of biological processes enriched in NSCLC resistant to EGFR-TKIs was built using Pathway Enrichment Analysis ClueGO (version 2.3.2) (Bindea et al., 2009).

### Survival Analysis Based on the ceRNA Regulatory Network

To identify associations between the expression of nodes in the ceRNA network and overall survival of patients, Kaplan‐Meier survival analysis was performed using the ‘survfit’ function in the ‘survival’ package in R (https://CRAN.Rproject.org/package=survival) (Xu et al., 2014). We also screened ceRNA regulatory sub-networks of interacting lncRNAs, miRNAs and mRNAs in the TCGA data in order to identify those significantly associated with survival (*p* < 0.05).

### Cell Culture

The NSCLC cell lines PC9, sensitive to Gefitinib, and PC9-GR, resistant to Gefitinib, were obtained from Heilongjiang Cancer Institute (Harbin, China). As described, an EGFR-TKI resistant cell line (PC9-GR) were established by treating EGFR-TKI-sensitive PC9 cell with continuous gefitinib culture (Huang et al., 2020b). These cells were cultured in RPMI 1640 medium (Gibco; Thermo Fisher Scientific, Inc.) supplemented with 10% fetal bovine serum (Gibco; Thermo Fisher Scientific, Inc.) and 1% penicillin-streptomycin at 37°C in a humidified atmosphere of 5% CO_2_.

### Quantitative Real-Time PCR

This quantitative real-time PCR analysis was conducted as described using the following primers, all synthesized by Sangon (Shanghai, China) (Wang et al., 2019a): ABTB1, 5′-GGC​GGG​ATT​ACT​ATG​ACG​AC-3′ (forward) and 5′-GCC​TGA​GAA​CCA​CGA​CAC​TC-3′ (reverse); NPTN, 5′-GCT​CCT​AAA​GCA​AAC​GCC​ACC​A-3′ (forward) and 5′-TCT​TGC​GCC​ATA​TCC​AGT​CTG​G-3′ (reverse); SERPINE1, 5′-CTC​ATC​AGC​CAC​TGG​AAA​GGC​A-3′ (forward) and 5′-GAC​TCG​TGA​AGT​CAG​CCT​GAA​AC-3′ (reverse); and hsa-miR-150-5p-R, 5′-CCG​TCT​CCC​AAC​CCT​TGT​AC-3′ (forward) and 5′-CAG​TGC​AGG​GTC​CGA​GGT-3′ (reverse).

### Cell Viability Assay

Cell viability assay was performed as previously demonstrated (Wang et al., 2019a). The cells were seeded in 96-well plates with 2*10^3^ cells per well with or without tiplaxtinin (40 μm) (HY-15253, MedChem Express) incubated for 24 h before seeding on the 96-well culture plate. Subsequently, gefitinib was added to the wells incubating for 48 h at the indicated concentrations. After gefitinib treatment, the viability of cells was measured.

### Statistical Analysis

All statistical analyses were carried out using SPSS 24.0 software (IBM Corp, Armonk, NY, United States ) and GraphPad Prism software 8.0 (GraphPad Software, San Diego, CA, United States). Data were expressed as mean ± standard deviation. Differences between samples resistant or sensitive to EGFR-TKIs were assessed for significance using Student’s t test. The ability of the core ceRNA networks to predict resistance to EGFR-TKI was assessed based on the area under curves (AUCs) of the receiver operating characteristic (ROC) curves. The AUC is obtained by summing the areas of the parts under the ROC curve. Survival was analyzed using the Kaplan-Meier approach, and survival was compared between groups using the log-rank test. Differences were considered significant if they were associated with a two-tailed *p* < 0.05.

## Results

### Expression of lncRNAs, miRNAs, and mRNAs in NSCLC

**FIGURE 1:**
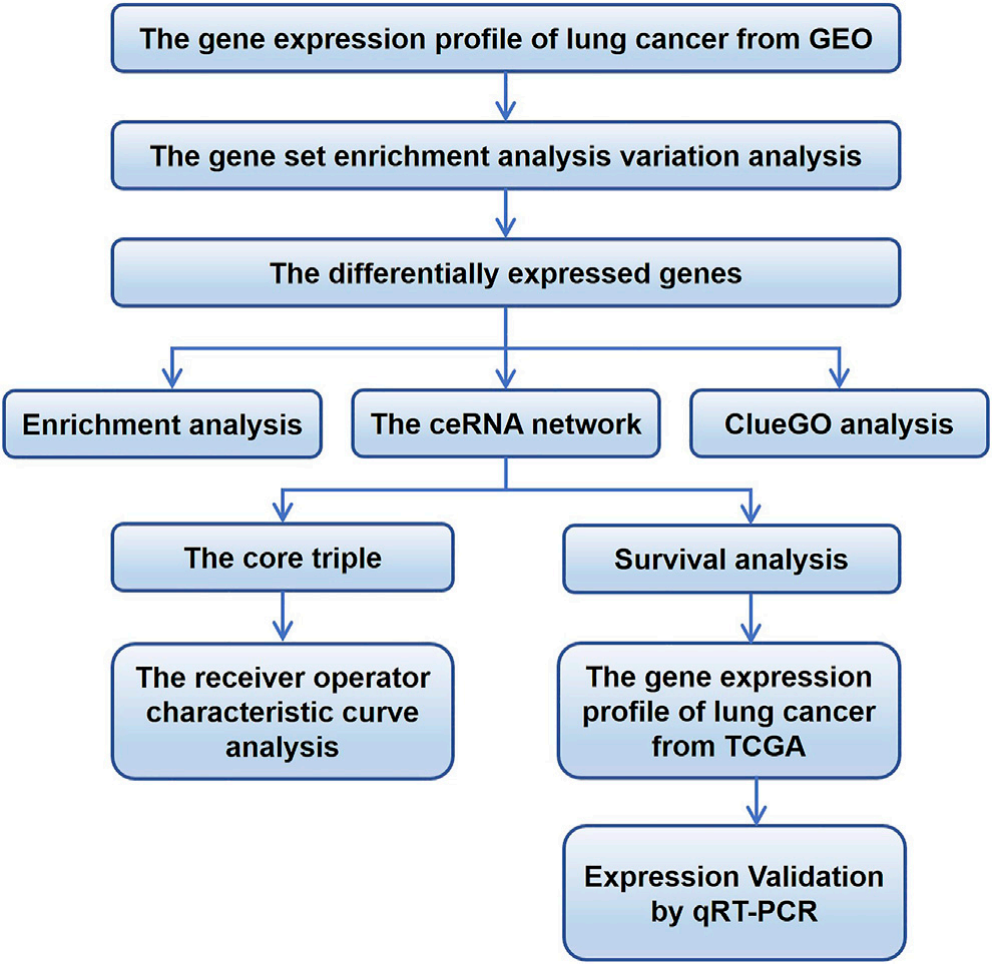
Flowchart of the study. ceRNA, competing endogenous RNA; GEO, Gene Expression Omnibus; TCGA, The Cancer Genome Atlas.

**FIGURE 2:**
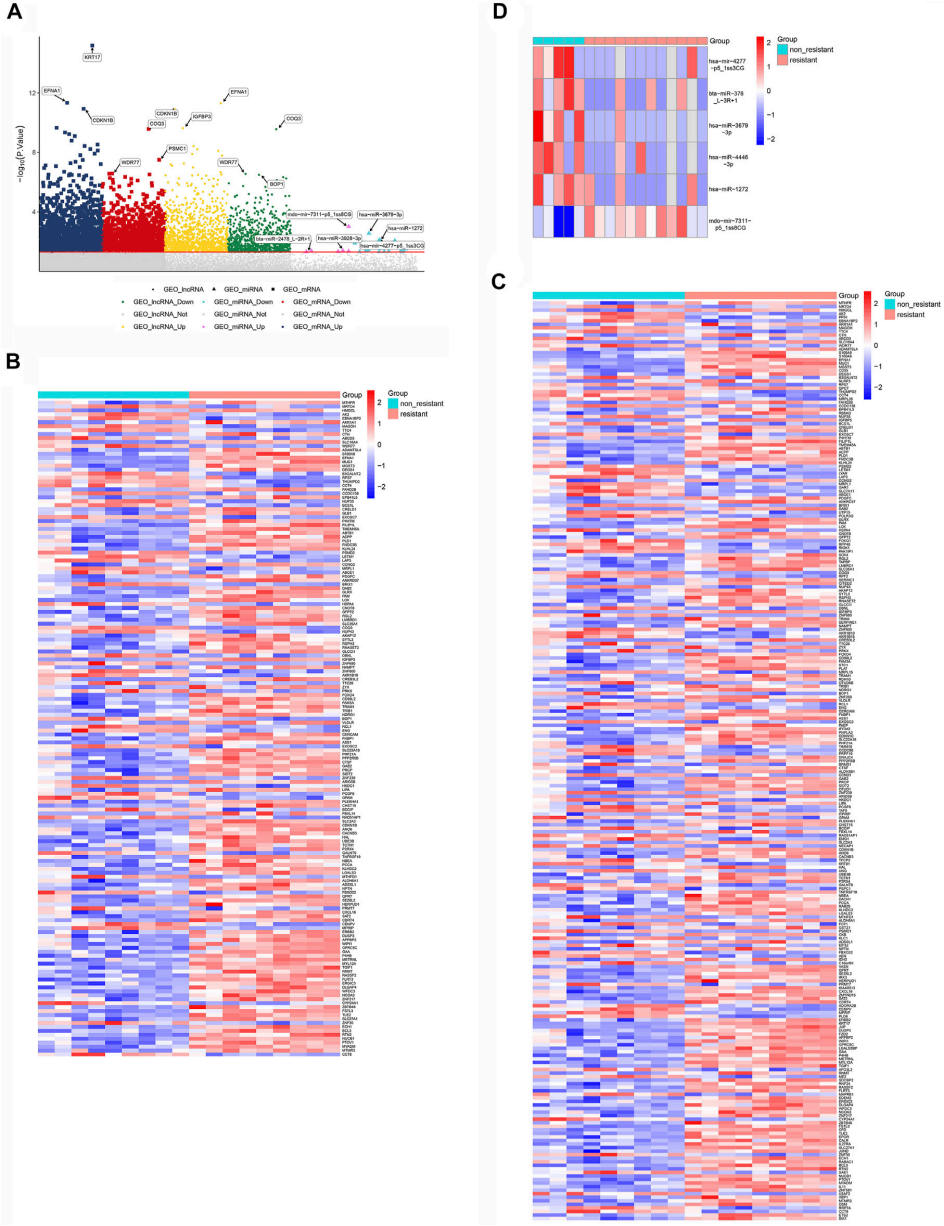
Differentially expressed lncRNAs, miRNAs and mRNAs potentially associated with NSCLC resistance to EFRG-TKIs. **(A)** Manhattan chart, showing lncRNAs, mRNAs and miRNAs along the *x*-axis, and log_10_(*p* value) for each gene along the *y*-axis. The gray dots below the dividing line indicate non-differentially expressed genes, while dots of other colors are differentially expressed genes. The significantly down- and up-regulated differentially expressed RNAs are depicted with different colors. **(B)** Differentially expressed lncRNAs. **(C)** Differentially expressed mRNAs. **(D)** Differentially expressed miRNAs.

Expression profiles for NSCLC cells sensitive or resistant to EGFR-TKI were acquired from the GEO databases, then standardized and analyzed using limma. The flow chart summarizing the framework is exhibited in Figure 1. The GSE83666 and GSE75309 databases were combined and divided into resistant group and non-resistant group. 1755 up-regulated mRNAs, 1,436 down-regulated mRNAs, up-regulated 4,598 lncRNAs and 4,391 down-regulated lncRNAs were screened with *p* value <0.05. GSE103352 data set was divided into resistant and non-resistant groups. 505 up-regulated miRNAs and 578 down-regulated miRNAs were screened with *p* value <0.05. DEGs were depicted in a Manhattan chart (Figure 2A) and a heatmap (Figures 2B–D), revealing 6,858 genes that were upregulated in resistant cancer, comprising 1,755 mRNAs, 4,598 lncRNAs, and 505 miRNAs; as well as 6,405 downregulated genes, comprising 1,436 mRNAs, 4,391 lncRNAs, and 578 miRNAs.

### DEG Enrichment in GO Processes and KEGG Pathways

**FIGURE 3:**
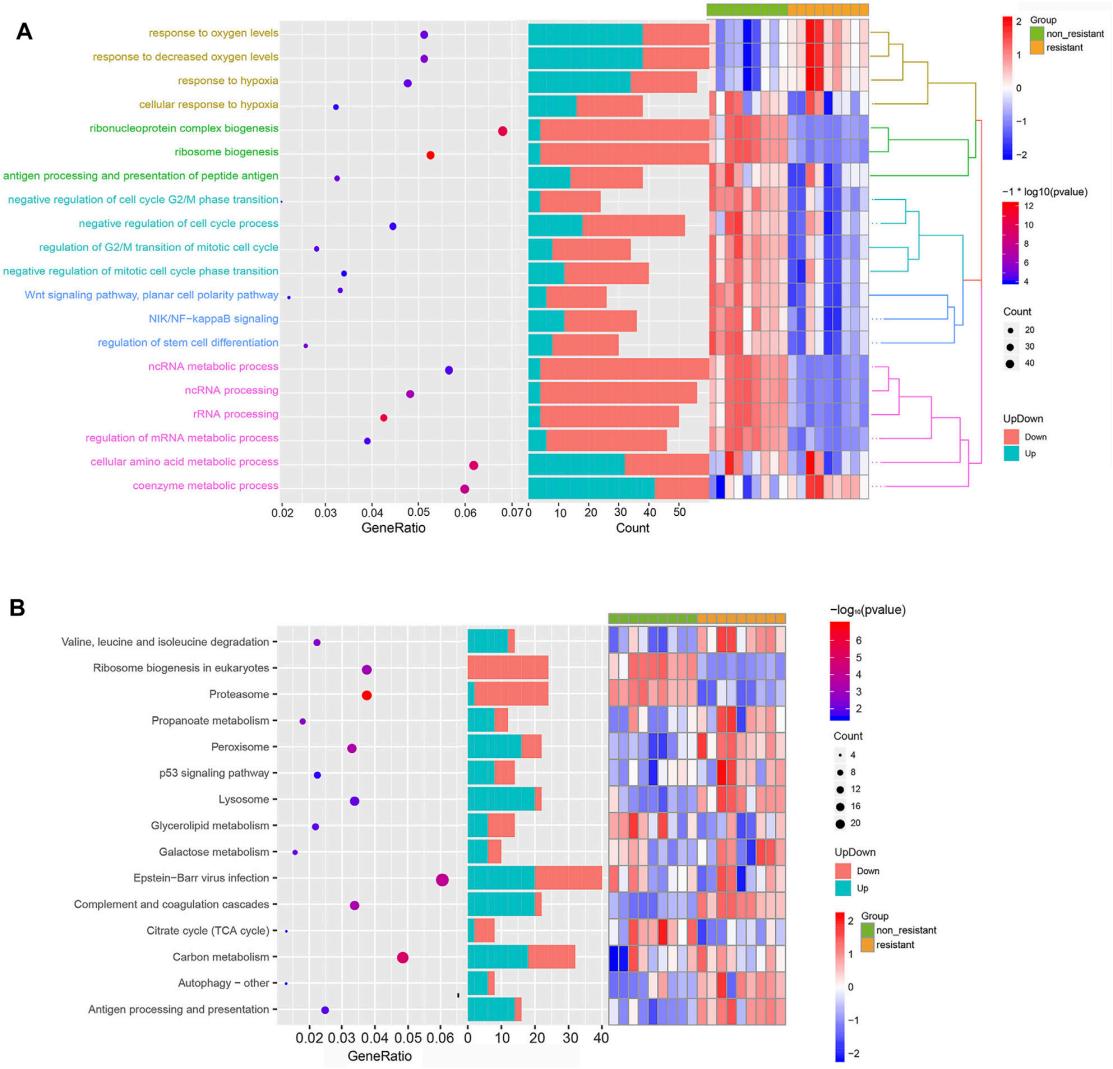
Functional enrichment of DEGs related to NSCLC resistance to EGFR-TKIs. **(A)** Top 20 significant GO biological processes. **(B)** Top 15 significant KEGG pathways.

**FIGURE 4:**
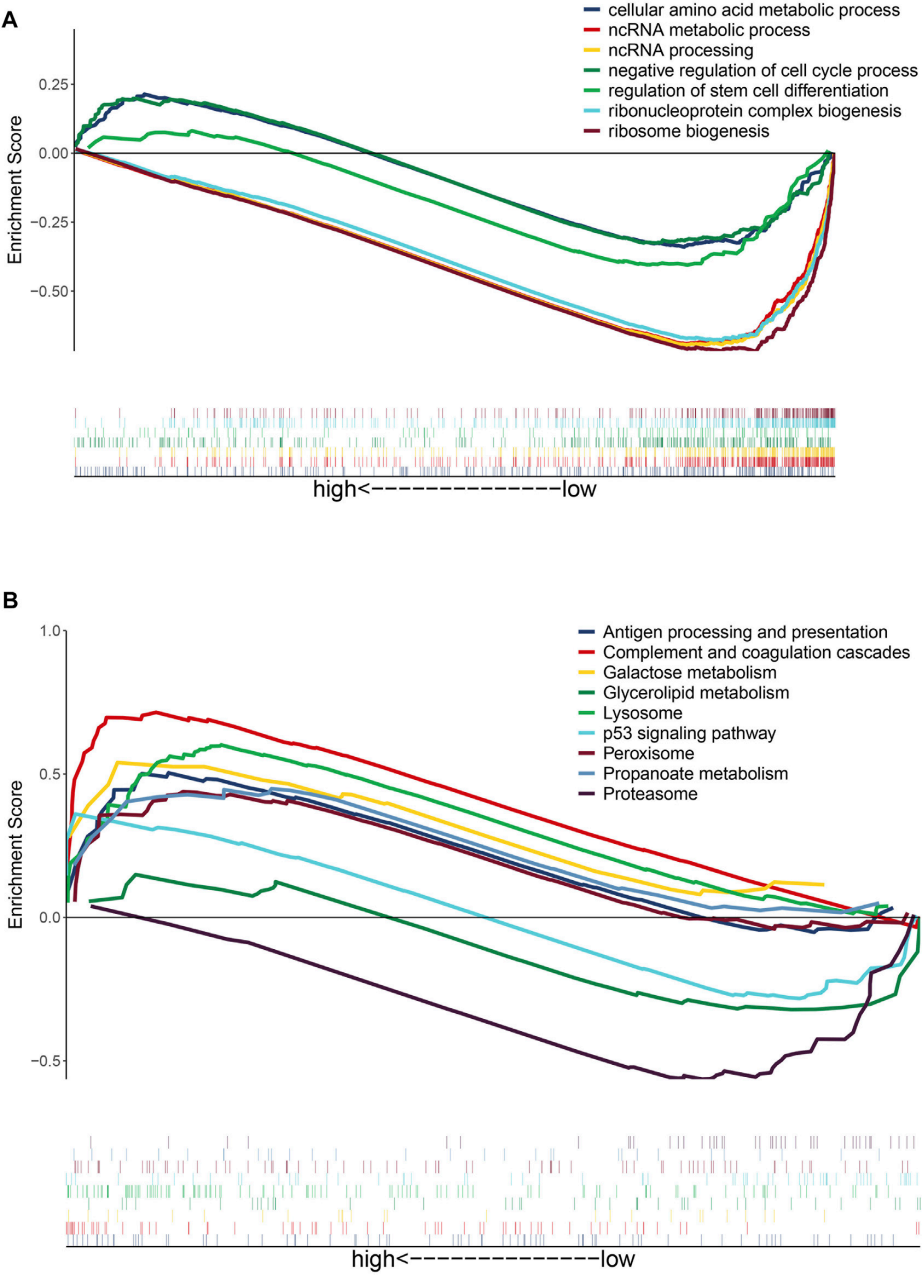
Gene set enrichment analysis of differentially expressed genes potentially related to NSCLC resistance to EGFR-TKIs. **(A)** GO biological processes of candidate hallmarks. **(B)** KEGG pathways of candidate hallmarks.

To gain insights into the function of DEmRNAs identified in the GSE83666 and GSE75309 datasets, we analyzed their enrichment in GO processes and KEGG pathways. A total of 968 biological processes and 31 KEGG pathways were identified with the filter criteria of adjust *p*-value< 0.05, of which 20 biological processes and 15 KEGG pathways have previously been linked to lung cancer resistance to EGFR-TKIs (Figures 3A,B). In addition, Gene Set Enrichment Analysis (GSEA) software was applied to identify GO-BP and KEGG pathway of the candidate hallmarks (Subramanian et al., 2005). GSEA identified seven biological processes and nine KEGG pathways significantly involved in resistance to EGFR-TKIs, including cellular amino acid metabolic processes, p53 signaling and proteasome pathways (Figures 4A,B). A potential network of biological processes enriched in NSCLC resistant to EGFR-TKIs was built using Pathway Enrichment Analysis ClueGO (Supplementary Figure S1).

### Construction of a Transcriptional Regulatory Network Linking lncRNAs, miRNAs, and mRNAs

**FIGURE 5:**
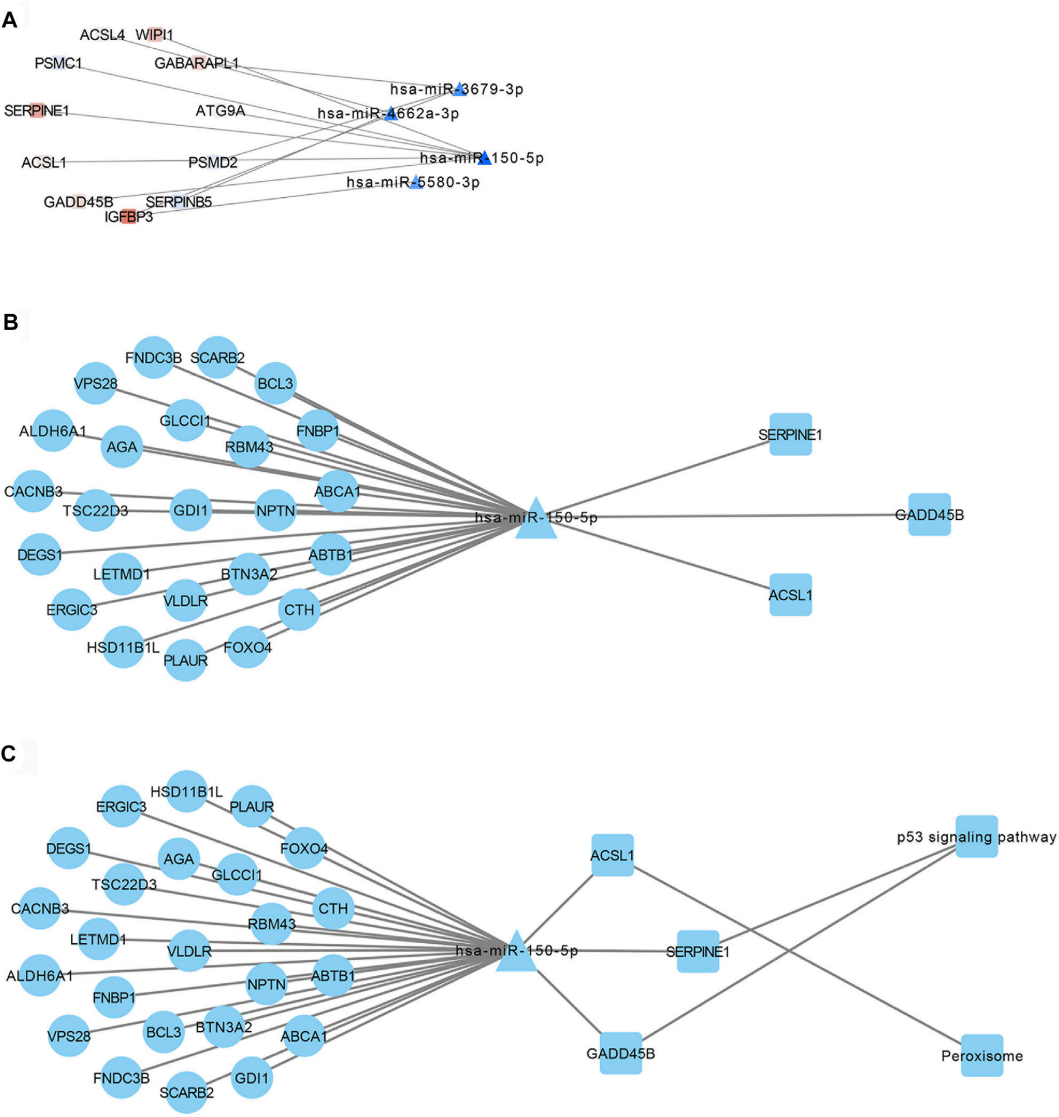
Transcriptional regulatory network of lncRNAs, miRNAs and mRNAs involved in NSCLC resistance to EGFR-TKIs. **(A)** Interactions between miRNAs and mRNAs involved in such resistance. **(B)** Identification of ceRNA sub-networks, based on the criteria R > 0.75 and *p* < 0.01. **(C)** Combination of the transcriptional regulatory network with KEGG pathways.

We constructed a ceRNA regulatory network linking lncRNAs, miRNAs and mRNAs by identifying miRNA–mRNA interactions (Figure 5A) and lncRNA–miRNA interactions (Supplementary Figure S2). The correlation function in Hmisc package was used for correlation analysis, and 329 ceRNA sub-networks were obtained. Pearson correlation analysis of lncRNAs and mRNAs in the network were screened for those satisfying the criteria of R > 0.75 and *p* < 0.01, leading to 31 ceRNA regulatory sub-networks (Figure 5B). When enriched KEGG pathways were added to the network, p53 signaling and peroxisome pathways emerged as potentially linked to resistance (Figure 5C). In this way, our study provides valuable leads for future experiments to explore the molecular mechanisms of NSCLC resistance to EGFR-TKIs.

### Correlations Among ceRNAs and Their Association With Survival

**FIGURE 6:**
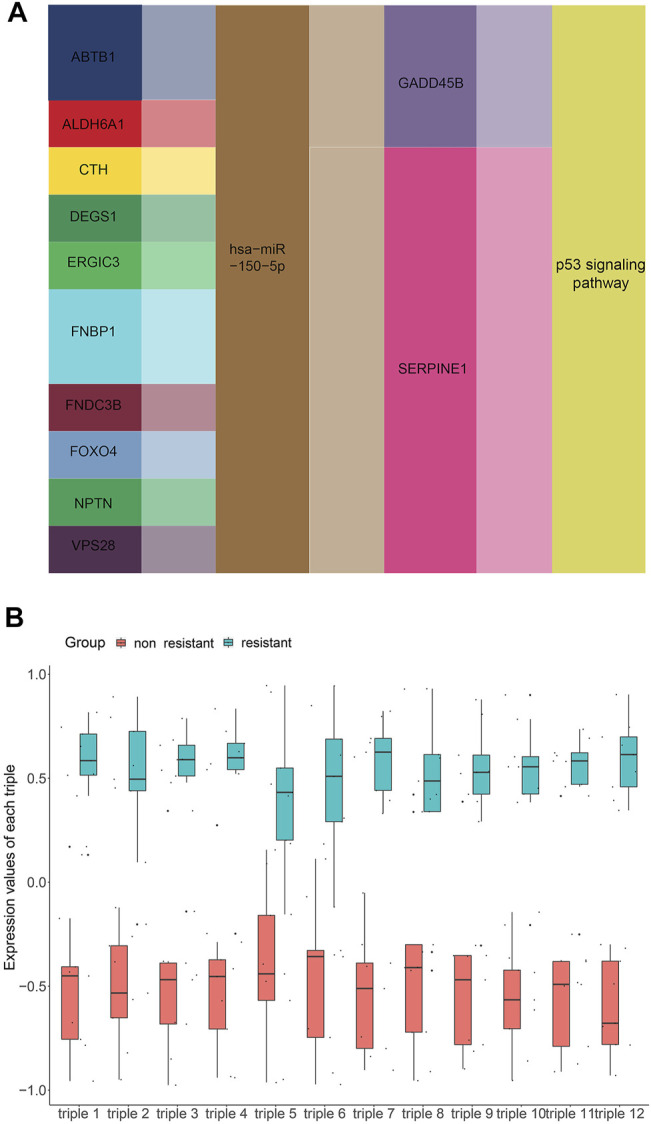
Screening for core lncRNA-miRNA-mRNA networks. **(A)** Identification of core lncRNA-miRNA-mRNA networks, based on the criteria R > 0.85 and *p* < 0.01. **(B)** Expression of core lncRNA-miRNA-mRNA networks in non-resistant group and EGFR-TKIs resistant group.

To get the core ceRNA sub-networks, we screened these pathways for those satisfying the criteria R > 0.85 and *p* < 0.01, identifying 12 core ceRNA regulatory sub-networks (Figure 6A; Table 1). These core ceRNA regulatory sub-networks (triple 1–12) were expressed at significantly different levels between sensitive or resistant samples (Figure 6B). The ability of these regulatory sub-networks to predict resistance was assessed based on AUCs. Triple 3, 7, 9, 10 and 12 showed better predictive performance than other ceRNA regulatory sub-networks (Supplementary Figure S3).

**TABLE 1:** 12 core ceRNA regulatory pahyways associated with resistance to EGFR-TKIs in NSCLC.

Triple ID	lncRNA	miRNA	mRNA
1	ERGIC3	hsa-miR-150–5p	SERPINE1
2	DEGS1	hsa-miR-150–5p	SERPINE1
3	ALDH6A1	hsa-miR-150–5p	SERPINE1
4	VPS28	hsa-miR-150–5p	SERPINE1
5	FNDC3B	hsa-miR-150–5p	GADD45B
6	FNBP1	hsa-miR-150–5p	GADD45B
7	FNBP1	hsa-miR-150–5p	SERPINE1
8	ABTB1	hsa-miR-150–5p	GADD45B
9	ABTB1	hsa-miR-150–5p	SERPINE1
10	CTH	hsa-miR-150–5p	SERPINE1
11	FOXO4	hsa-miR-150–5p	SERPINE1
12	NPTN	hsa-miR-150–5p	SERPINE1

**FIGURE 7:**
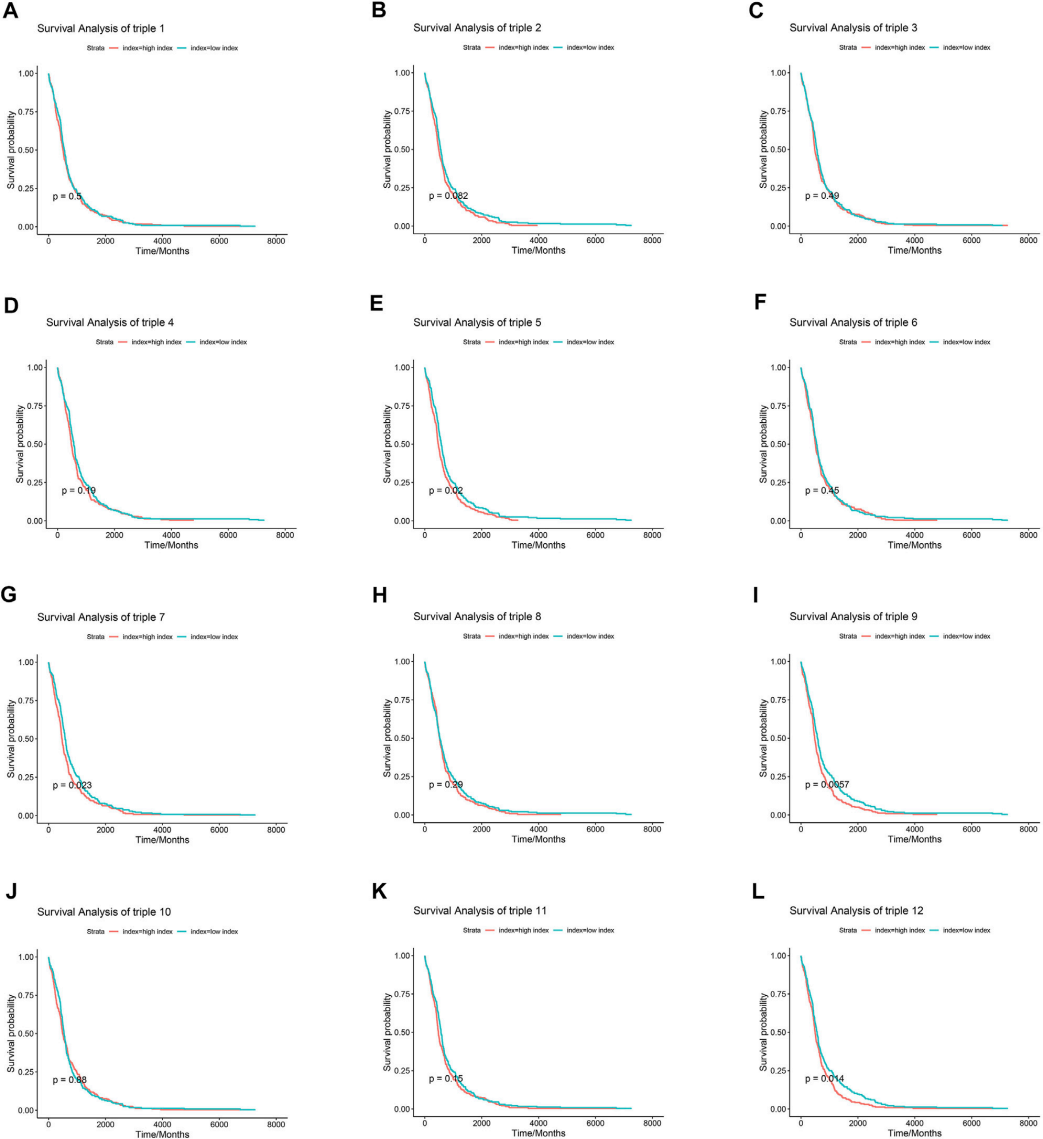
Association between core lncRNA-miRNA-mRNA networks and progression-free interval (PFI) in patients with NSCLC. Red lines show the high-expression group; green lines, the low-expression group. The horizontal axis demonstrates the PFI time in years, and the vertical axis indicates the survival rate.

**FIGURE 8:**
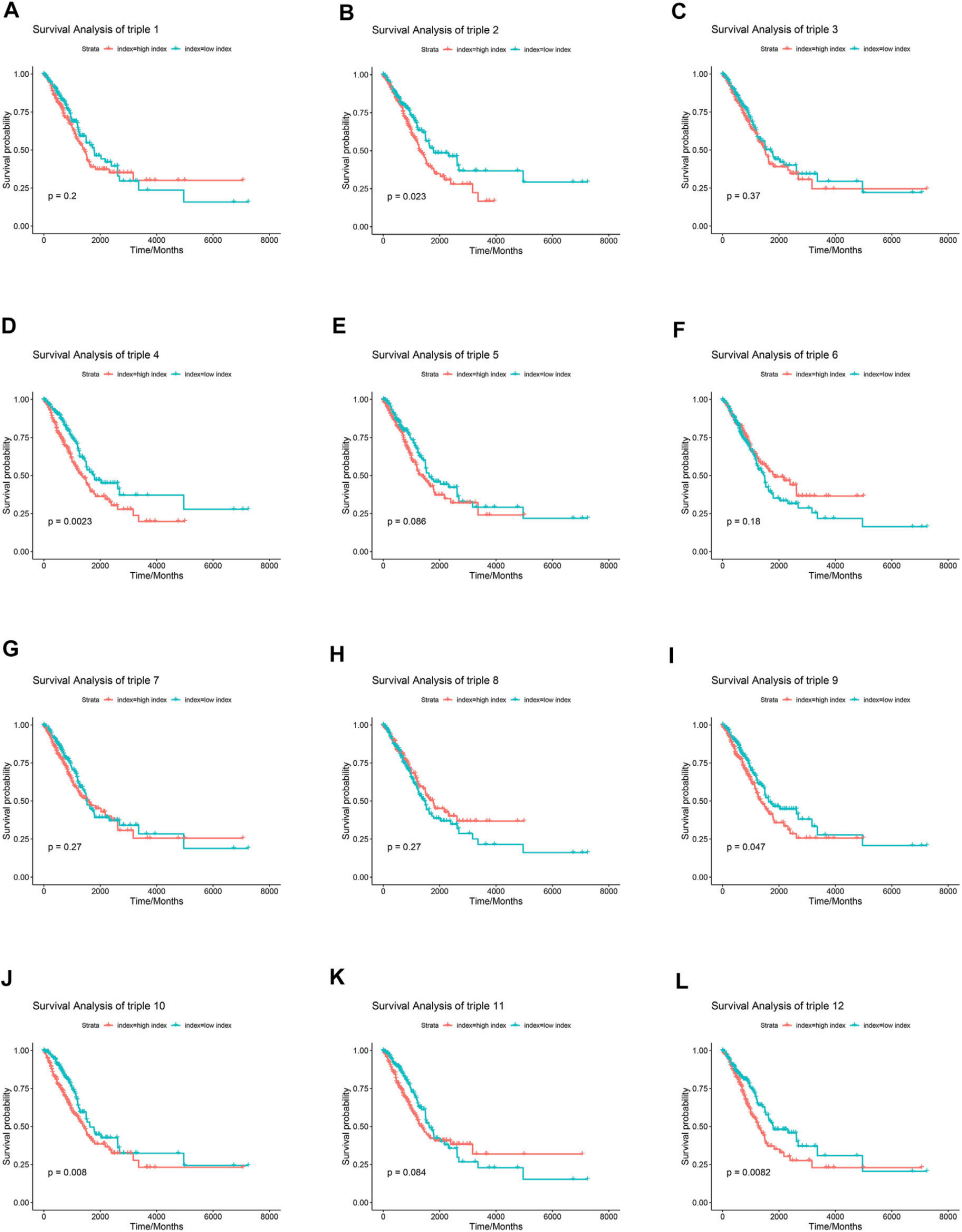
Association between core lncRNA-miRNA-mRNA regulatory sub-networks and Overall survival (OS) in patients with NSCLC in the TCGA. Red lines indicate the higher expression group. Greened lines indicate lower expression group. The horizontal axis demonstrates the overall survival time in years, and the vertical axis indicates the survival rate.

Numerous researches have reported that ceRNA may be involved in EGFR-TKI resistance, and may influence the progression of EGFR-mutant NSCLC. To examine whether ceRNAs are related to prognosis of NSCLC patients carrying EGFR mutations, Kaplan-Meier analysis and Cox regression modeling were performed based on TCGA data on progression-free interval (PFI) and overall survival (OS). Triple 9 and 12 were associated with PFI and OS (Figures 7, 8).

In addition, the Kaplan-Meier plotter online tool (http://kmplot.com/analysis/) was used to verify the effect of SERPINE1 on the survival of lung cancer. As shown in Supplementary Figure S4, the lung cancer patients with elevated SERPINE1 expression levels had poor OS than patients with corresponding low expression levels (*p* < 0.001). Consistently, as demonstrated in Supplementary Figure S5, high SERPINE1 expression predicts a poor prognosis in NSCLC using the web-based tools in The Human Protein Atlas (https://www.proteinatlas.org) based on the TCGA database.

### Validation of Representative Core CeRNA Regulatory Sub-networks in Human NSCLC Cell Lines

**FIGURE 9:**
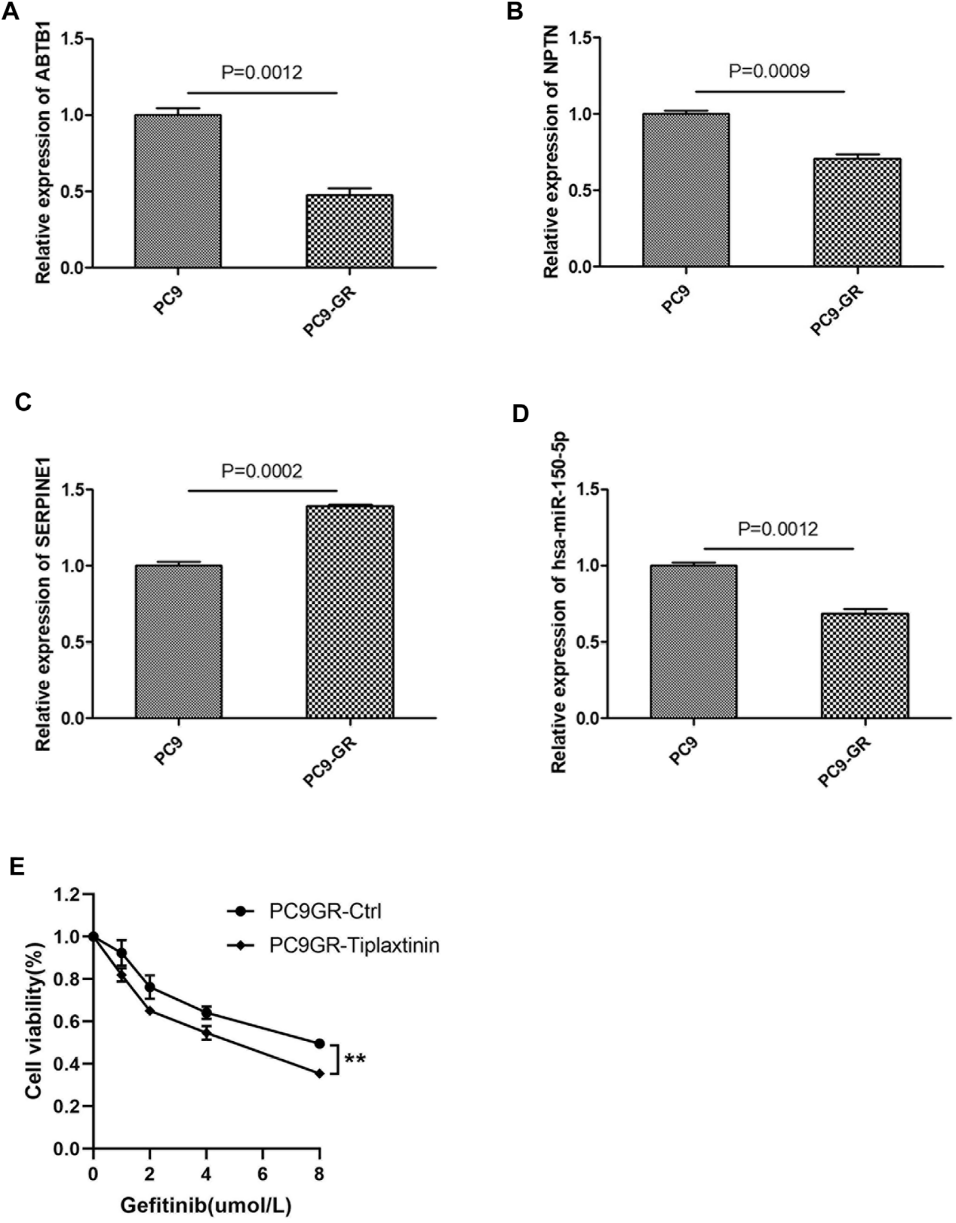
Validation of representative core ceRNa regulatory sub-networks in human NSCLC cell lines. **(A–D)** Quantitative real-time PCR validation of the dysregulation of ABTB1, NPTN, hsa-miR-150-5p and SERPINE1 expression in NSCLC cell lines sensitive to EGFR-TKIs (PC9) or resistant to EGFR-TKIs (PC9-GR). **(E)** PC9GR and PC9GR-Tiplaxtinin ( a inhibitor of SERPINE1) cells were treated with different concentrations of gefitinib, and cell viability assay was performed when the cells were cultured with gefitinib for 48 h.

To verify the reliability of the bioinformatics results, we investigated the expression of the core ceRNA triple 9 and 12 using qRT-PCR in NSCLC cell lines. Expression of the two lncRNAs ABTB1 and NPTN as well as the hsa-miR-150–5p was lower in the PC9-GR cell line than in the PC9 line (Figures 9A–D). Conversely, expression of the mRNA SERPINE1 was higher in the PC9-GR cell line than in the PC9 line. These results were consistent with the bioinformatics analysis. As shown in Figure 9E, inhibition of SERPINE1 can affect the sensitivity of EGFR-TKI drug-resistant cells to gefitinib at different concentrations.

## Discussion

NSCLC is an important factor in the incidence and mortality of carcinoma in human (1). Despite EGFR amplification may be predictive of the favorable response to EGFR-TKI, acquired resistance develops eventually (Yao et al., 2010). Therefore, more researches need to be performed to further elucidate the mechanism of EGFR-TKIs resistance. Our work supports several previous studies suggesting that mRNAs, miRNAs, and lncRNAs play important roles in NSCLC resistance to EGFR-TKIs (Li et al., 2018; Chen et al., 2020a; Huang et al., 2020a; Shu et al., 2020). We constructed lncRNA-miRNA-mRNA ceRNA networks that predicts numerous pathways that may mediate such resistance. We further identified nodes in these ceRNA networks that are associated with PSI and OS.

It is common knowledge that, most of the functions of ncRNA are achieved by regulating the expression of some key mRNAs. And, many studies have carried out a comprehensive analysis of ncRNA and its related co-expression and ceRNA network differential expression profiles in NSCLC. Yu and Ren (2021) constructed a ceRNA network by analyzing the differentially expressed lncRNAs of NSCLC transcriptome profiling from TCGA. The network can effectively predict the overall survival of NSCLC and may guide the treatment of NSCLC. Li et al. (2020) identified and integrated analysis of DEGs associated with prognosis in NSCLC, and constructed a ceRNA network for the study of NSCLC. Wang X et al. (Wang et al., 2019b) recognized differently expressed genes, and provided a lncRNA-related ceRNA networks in NSCLC. However, investigations on the molecular regulation network of lncRNA–miRNA–mRNA underlying EGFR-TKIs resistant is seldom. By focusing on resistance to EGFR-TKIs, our results extend previous studies that examined DEGs that may predict prognosis in NSCLC. We found that DEGs in resistant cancer tissue may be involved in ribosome biogenesis, proteasome activity and metabolic pathways.

Based on bioinformatics analysis of DEGs linked to EGFR-TKI resistance, we built a corresponding lncRNA-miRNA-mRNA regulatory network, and we identified 12 core ceRNA regulatory sub-networks. We discovered that two groups of lncRNA-miRNA-mRNA interactors were significantly associated with NSCLC resistance to EGFR-TKIs as well as with patient survival. We validated the dysregulation of these two groups of interactors in human NSCLC cell lines sensitive or resistant to EGFR-TKIs.

The “ceRNA hypothesis” (Salmena et al., 2011) describes how mRNAs, transcriptional pseudogenes and lncRNAs “talk” to one another via shared miRNAs, and this hypothesis has proven useful for explaining many cancer pathways. For example, the lncRNA UCA1 acts via the miR-143/FOSL2 axis to modulate gefitinib resistance in NSCLC (Chen et al., 2020b). The lncRNA KCNQ1OT1 acts via miR-211–5p and downstream Ezrin/Fak/Src signaling to promote tumor growth and cisplatin resistance in tongue cancer (Zhu et al., 2021). The lncRNA HOXA11-AS acts via miR-454–3p and Stat3 to promote cisplatin resistance in lung adenocarcinoma (Zhao et al., 2018). Our results provide support that ceRNA regulatory sub-networks play a key role in NSCLC and its development of resistance to EGFR-TKIs.

## Conclusion

As far as we know, this is the first comprehensive expression profiling of non-coding RNAs and of mRNAs potentially linked to EGFR-TKI resistance in NSCLC cells. We built a ceRNA network that provides numerous testable hypotheses about the processes and pathways that give rise to such resistance.

## Data Availability

Publicly available datasets were analyzed in this study. This data can be found here: GSE83666, GSE75309, GSE103352.

## Abbreviations

ceRNA, competitive endogenous RNA; DE, differently expressed; EGFR, Epidermal growth factor receptor; EGFR-TKI, EGFR tyrosine kinase inhibitors; GO, Gene Ontology; GEO, Gene Expression Omnibus; GO-BP, Gene Ontology Biological process; KEGG, Kyoto Encyclopedia of Genes and Genomes; LncRNA, Long noncoding RNA; ncRNAs, non-coding RNAs; NSCLC, non-small cell lung cancer; OS, overall survival; miRNAs, microRNAs; PFI, progression-free interval; qRT-PCR, quantitative real-time reverse transcription-polymerase chain reaction; TCGA, The Cancer Genome Atlas.
