# Supplementary figures and images for: Identification of lncRNA–miRNA–mRNA Networks Linked to Non-small Lung Cancer Resistance to Inhibitors of Epidermal Growth Factor Receptor
Source: Front Genet. 2021 Nov 12;12:758591. doi: 10.3389/fgene.2021.758591 (PMC8632870; doi:10.3389/fgene.2021.758591)

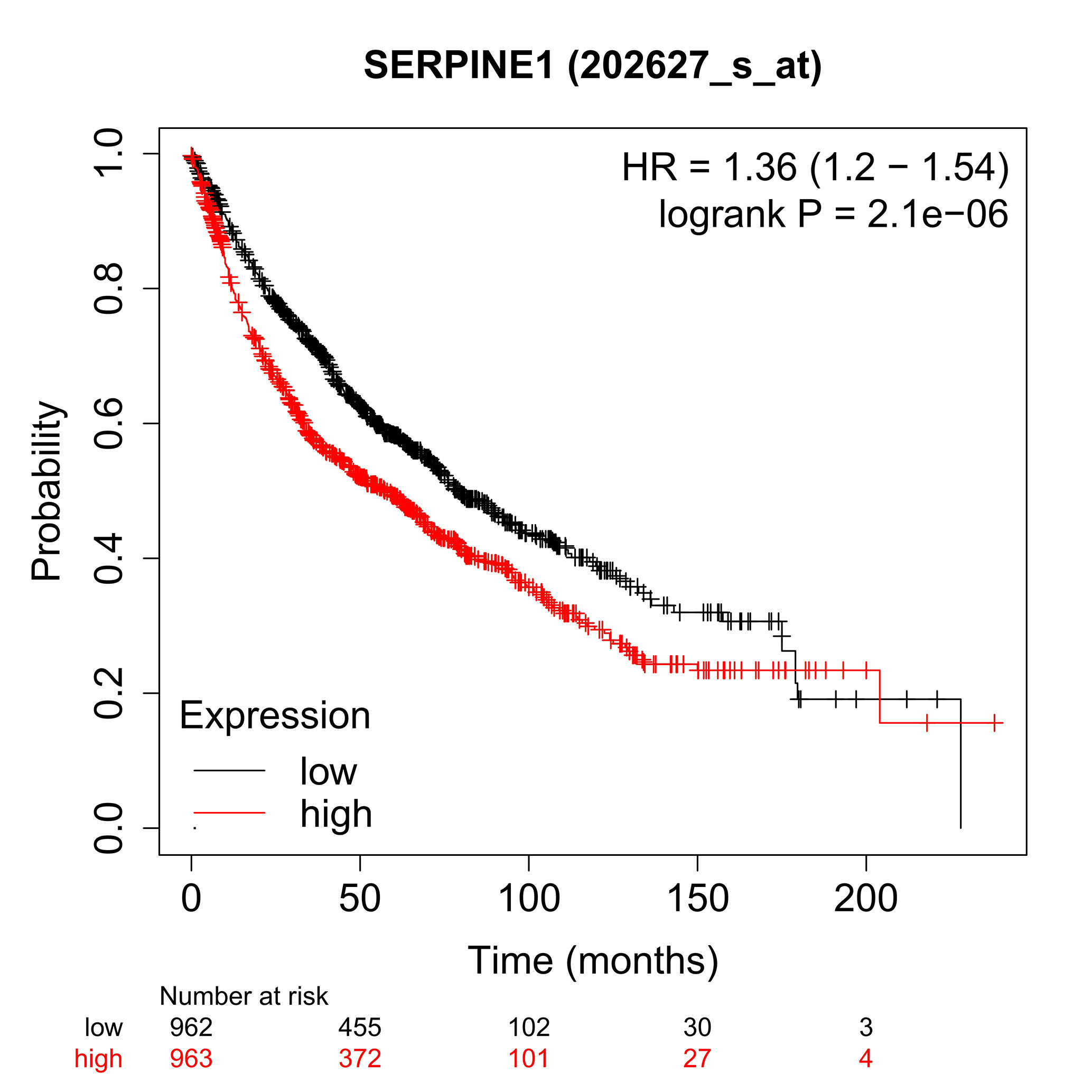

Supplement: Supplementary file 2 [file Image4.JPEG]

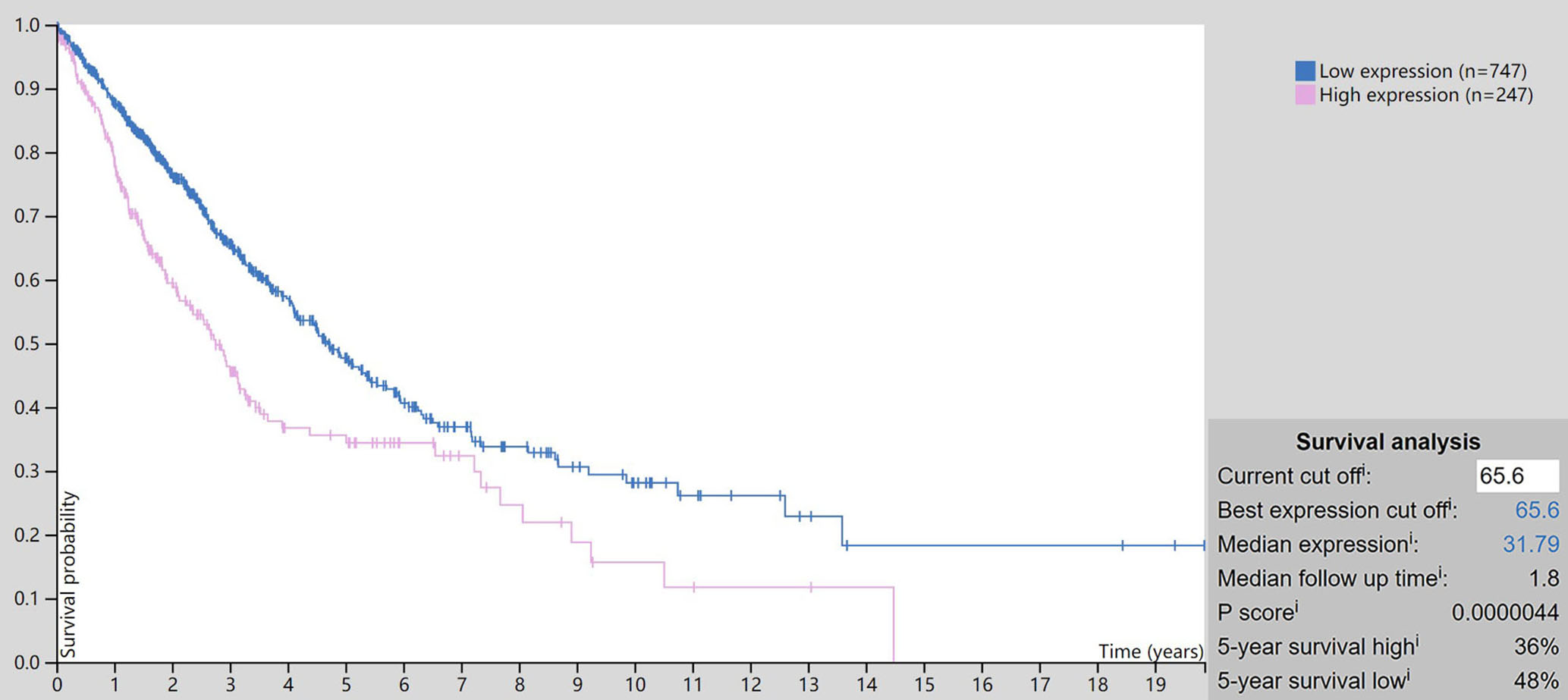

Supplement: Supplementary file 4 [file Image5.JPEG]
