# Supplementary material for: Identification of lncRNA–miRNA–mRNA Networks Linked to Non-small Lung Cancer Resistance to Inhibitors of Epidermal Growth Factor Receptor
Source: Front Genet. 2021 Nov 12;12:758591. doi: 10.3389/fgene.2021.758591 (PMC8632870; doi:10.3389/fgene.2021.758591)
Supplement: Supplementary file 6 [file DataSheet1.docx]

**Supplementary Figure 1.** The ceRNA network of all differentially expressed mRNAs in NSCLC tissues resistant to EFRG-TKIs, generated using ClueGO.

**Supplementary Figure 2.** Network of lncRNAs, miRNAs, mRNAs, and KEGG networks associated with NSCLC resistance to EGFR-TKIs, generated using the RNAInter database.

**Supplementary Figure 3.** Areas under the receiver operating characteristic curve (AUCs) for all core lncRNA-miRNA-mRNA networks.

**Supplementary Figure 4.** High SERPINE1expression is associated with poor prognosis in lung cancer patients (n=1925, P<0.001). The hazard ratio (HR) and the log-rank test were used in the survival analysis.

**Supplementary Figure 5.** High SERPINE1 expression is associated with poor prognosis in NSCLC patients (n=994, P<0.001) from the TCGA database.
